# Clinical Significance and Effect of lncRNA HOXA11-AS in NSCLC: A Study Based on Bioinformatics, *In Vitro* and *in Vivo* Verification

**DOI:** 10.1038/s41598-017-05856-2

**Published:** 2017-07-17

**Authors:** Yu Zhang, Wen-jie Chen, Ting-qing Gan, Xiu-ling Zhang, Zu-cheng Xie, Zhi-hua Ye, Yun Deng, Ze-feng Wang, Kai-teng Cai, Shi-kang Li, Dian-zhong Luo, Gang Chen

**Affiliations:** 1 Department of Pathology, First Affiliated Hospital of Guangxi Medical University, 6 Shuangyong Road, Guangxi Zhuang Autonomous Region, Nanning, 530021 China; 2 Department of Thoracic and Cardiovascular Surgery, First Affiliated Hospital of Guangxi Medical University, 6 Shuangyong Road, Guangxi Zhuang Autonomous Region, Nanning, 530021 China; 3 Department of Medical Oncology, First Affiliated Hospital of Guangxi Medical University, 6 Shuangyong Road, Guangxi Zhuang Autonomous Region, Nanning, 530021 China

**Keywords:** Cancer, Cell biology, Materials science

## Abstract

HOXA11 antisense RNA (HOXA11-AS) has been shown to be involved in tumorigenesis and development of different cancers. However, the role of HOXA11-AS in non-small cell lung cancer (NSCLC) remains unclear. In this study, we firstly explored and confirmed the expression of HOXA11-AS in NSCLC tissues and cells. Cytometry, CCK-8, cell scratch, migration, Matrigel invasion and flow cytometry assays were performed to determine the biological impact of HOXA11-AS *in vitro*. Furthermore, a chick embryo chorioallantoic membrane (CAM) model of NSCLC was constructed to explore the effect of HOXA11-AS on tumorigenicity and angiogenesis *in vivo*. Additionally, bioinformatics analyses were performed to investigate the prospective pathways of HOXA11-AS co-expressed genes. As results, HOXA11-AS was markedly highly expressed in NSCLC tissues and cells. Furthermore, the proliferation, migration, invasion, tumorigenic and angiogenic ability of NSCLC cells were all inhibited and apoptosis was induced after HOXA11-AS knock-down. HOXA11-AS RNAi also led to cell cycle arrest on G0/G1 or G2/M phase. In addition, the non-small cell lung cancer pathway might be involved in regulating the co-expressed genes of HOXA11-AS in NSCLC. These results indicate that HOXA11-AS plays pivotal roles in NSCLC and it can become a novel therapeutic direction for treating NSCLC.

## Introduction

Lung cancer, which comprises small cell lung cancer (SCLC) and non-small cell lung cancer (NSCLC), is the predominant cause of cancer deaths globally, and the incidence rate ranks first among all malignancies^1–4^. NSCLC accounts for approximately 80% of lung cancer, and currently, it still has an unsatisfactory prognosis despite the advances made in its treatments, with an overall 5-year survival rate of less than 16%^5–7^. Therefore, a deeper understanding of the underlying molecular mechanisms related to NSCLC is of great importance.

Long non-coding RNAs (lncRNAs) are non-coding transcripts with length greater than 200 nucleotides that share similarities with messenger RNAs (mRNAs)^8^. They have no significant protein-coding capabilities but play an important part in the modification of various biological and pathological processes, such as tumor growth, metastasis, differentiation and immune response^9–12^. LncRNAs can also contribute to carcinogenesis or tumor suppression and play a role in regulating cell cycle, apoptosis, proliferation, invasion and migration through their aberrant expression in different cancers^11–13^. To date, the effect and mechanism of many lncRNAs in NSCLC has been reported. For example, MALAT-1 acts as an oncogene and is highly expressed in NSCLC tissues and cell lines. The migration and growth of tumors was inhibited after silencing MALAT-1 expression^14, 15^, whereas the forced expression of MALAT-1 significantly increased migration in NSCLC cells^14^. In addition, MALAT-1 directly regulated the expression of Bcl-2 to effect prognosis of NSCLC^16^. Some reports have revealed that lncRNAs play an important role in cancers via targeting miRNAs and regulating genes. For instance, PCA3 up-regulated the expression of PRKD3 by miR-1261 sponging to promote invasion and migration of prostate cancer cells^17^. Moreover, MALAT-1 inhibited proliferation, migration and invasion of esophageal squamous cell carcinoma cells via regulation of the expression of miR-101 and miR-217^18^.

LncRNA HOXA11 antisense RNA (HOXA11-AS), also identified as HOXA-AS5, HOXA11S, HOXA11-AS1, HOXA11AS, or NCRNA00076, is located on 7p15.2 with the NCBI Gene ID: 221883. HOXA11-AS is one of the homeobox (HOX) family genes, and the length of HOXA11-AS is 3885 nt. Interestingly, no HOXA11-AS expression was found in normal lung tissues (http://rna.sysu.edu.cn/deepBase/)^19^. Previously, we have investigated the expression and the underlying pathways of HOXA11-AS in NSCLC based on the Cancer Genome Atlas (TCGA) database and bioinformatics analyses (gene ontology (GO), pathway, Kyoto Encyclopedia of Genes and Genomes (KEGG), and network analyses)^20^. As results, we found that HOXA11-AS was significantly overexpressed in both lung adenocarcinoma and squamous cell carcinoma based on TCGA database. In addition, the high area under curve (AUC) of HOXA11-AS indicates a potential diagnostic value of HOXA11-AS level in NSCLC. Also, the bioinformatics analyses show that HOXA11-AS may play a significant role in development and progression of NSCLC via regulating various pathways and genes. However, the exact mechanism should be verified by functional experiments^20^. In this study, we aimed to explore the effect of HOXA11-AS on proliferation, migration, invasion and apoptosis of NSCLC *in vitro* and *in vivo*. The TCGA database was used to verify the differential expression of HOXA11-AS between normal lung and NSCLC tissues. Quantitative reverse transcription–polymerase chain reaction (qRT-PCR), lentivirus-mediated HOXA11-AS RNAi transfection, flow cytometry and CCK-8, cell scratch, migration, and Matrigel invasion assays were performed, as well as development of a chick embryo chorioallantoic membrane (CAM) model, to clarify the possible role of HOXA11-AS in NSCLC. In addition, Multi Experiment Matrix (MEM)^21, 22^ was used to analyze the potential pathways associated with HOXA11-AS.

## Results

### HOXA11-AS expression in NSCLC

**Figure 1:**
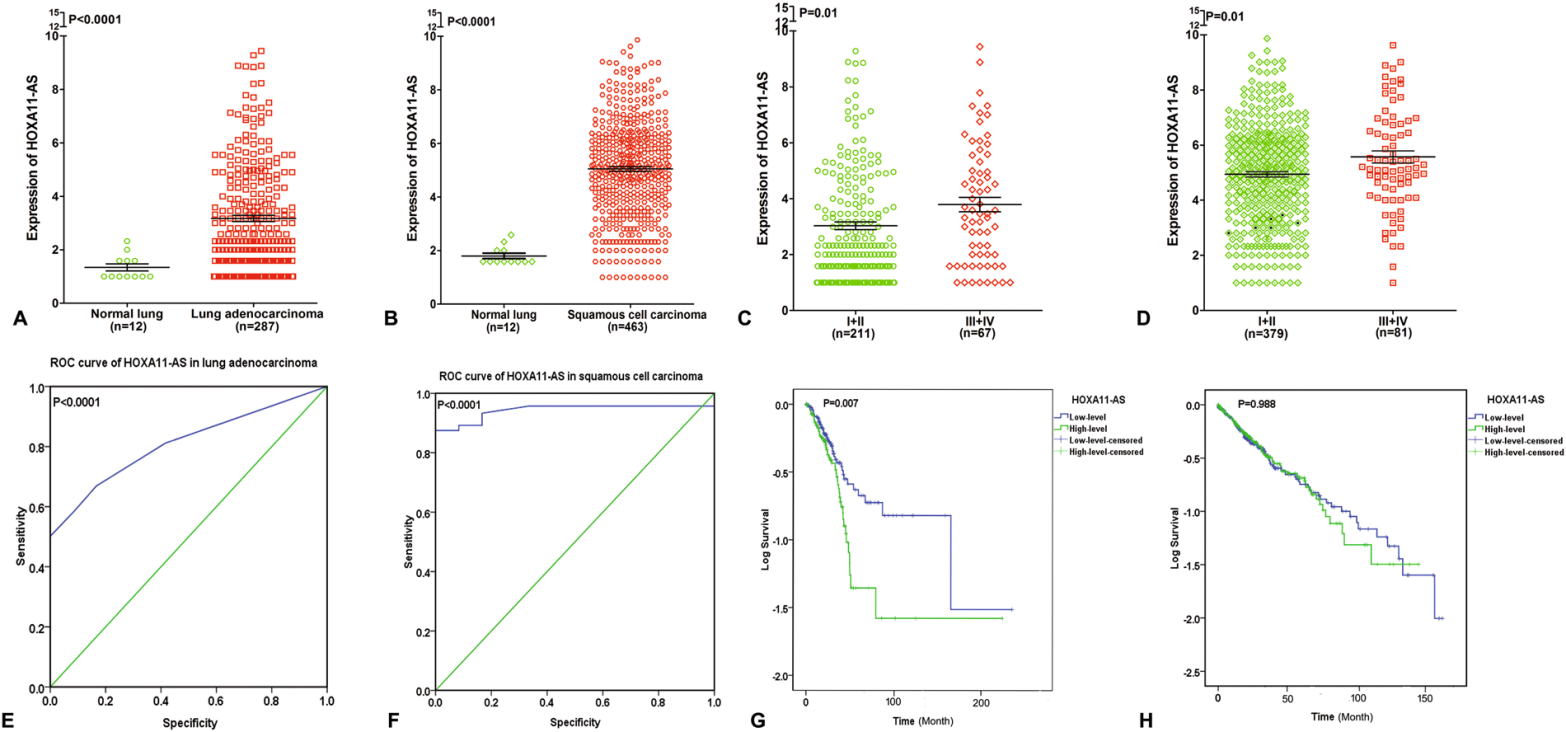
Differential expression and ROC curves of HOXA11-AS in lung adenocarcinoma and squamous cell carcinoma based on The Cancer Genome Atlas (TCGA) database. (**A**) Differential expression of HOXA11-AS in lung adenocarcinoma. (**B**) Differential expression of HOXA11-AS in squamous cell carcinoma. (**C**) Differential expression of HOXA11-AS in stage (I + II) and stage (III + IV) in lung adenocarcinoma. (**D**) Differential expression of HOXA11-AS in stage (I + II) and stage (III + IV) in lung squamous cell carcinoma. (**E**) ROC curve of HOXA11-AS in lung adenocarcinoma. (**F**) ROC curve of HOXA11-AS in lung squamous cell carcinoma. (**G**) Kaplan-Meier curves of HOXA11-AS expression in lung adenocarcinoma. (**H**) Kaplan-Meier curves of HOXA11-AS expression in lung squamous cell carcinoma.

**Figure 2:**
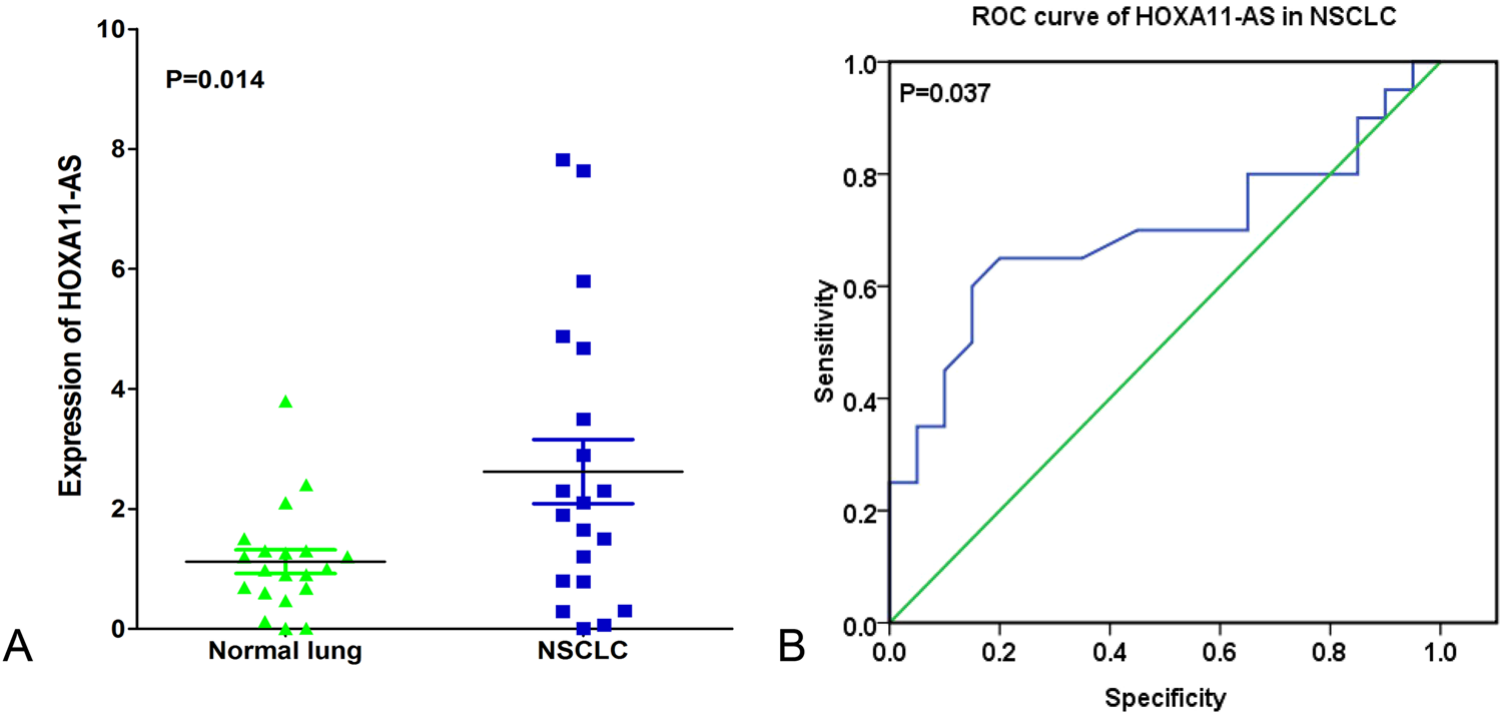
Differential expression and ROC curve of HOXA11-AS in NSCLC based on qRT-PCR. (**A**) Differential expression of HOXA11-AS in NSCLC. (**B**) ROC curve of HOXA11-AS in NSCLC.

To further explore the difference of HOXA11-AS expression between NSCLC compared with non-cancerous lung tissues, we performed clinical research using the original data in TCGA. Two NSCLC cohorts, which comprised lung adenocarcinoma patients (287 lung adenocarcinoma cases vs 12 non-cancerous lung cases) and lung squamous cell carcinoma patients (463 lung squamous cell carcinoma cases vs 12 non-cancerous lung cases) were extracted. Increased expression of HOXA11-AS was observed in lung adenocarcinoma tissues (3.173 ± 2.059) and lung squamous cell carcinoma tissues (5.049 ± 1.919) compared with the non-cancerous tissues (1.340 ± 0.466, 1.780 ± 0.349, respectively, both P < 0.001, Fig. 1A,B). With regard to the clinicopathological characteristics, we found that HOXA11-AS expression was higher (3.790 ± 2.143) in stage (III + IV) than in stage (I + II) (3.031 ± 2.025, P = 0.01, Fig. 1C) in lung adenocarcinoma as well as in lung squamous cell carcinoma (III + IV: 5.569 ± 1.980, I + II: 4.939 ± 1.896, P = 0.01, Fig. 1D). For the other clinicopathological parameters, no statistical associations were found based on TCGA database. Meanwhile, the area under the curve (AUC) of HOXA11-AS reached 0.811 (95% CI: 0.729–0.893, P < 0.0001, Fig. 1E) in lung adenocarcinoma and 0.952 (95% CI: 0.930–0.975, P < 0.0001, Fig. 1F) in lung squamous cell carcinoma, which indicate a high diagnostic value of the HOXA11-AS level in lung cancer. In addition, we investigated the prognostic significance of HOXA11-AS in the two cohorts of NSCLC patients in TCGA. We observed a trend in which low HOXA11-AS expression was correlated with a better survival (112.13 ± 16.09 months) compared to the high HOXA11-AS expression group (69.80 ± 11.32 months, P = 0.007, Fig. 1G) in lung adenocarcinoma, but no obvious trend was noted in lung squamous cell carcinoma (P = 0.988, Fig. 1H). Furthermore, an up-regulated trend in HOXA11-AS level in NSCLC tissues (2.621 ± 0.535) was found compared to corresponding non-cancerous lung tissues (1.121 ± 0.197, P = 0.014, Fig. 2A) based on qRT-PCR. In addition, the diagnostic value of the HOXA11-AS level in NSCLC was assessed by a receiver operating characteristic (ROC) curve and the AUC of HOXA11-AS was 0.692 (95% CI: 0.521–0.864, P = 0.037, Fig. 2B). We also explored the correlation between HOXA11-AS and gender or age, but no significant correlation was found in either lung adenocarcinoma or squamous cell carcinoma. This might partially be due to the limited sample size.

In addition, we also determined the expression level of HOXA11-AS among 4 NSCLC cell lines (PC9, A549, H460 and H1299). Consistent with the results in NSCLC tissues, an increased HOXA11-AS level was found in the 4 cell lines, especially in A549 cells (t = −22.242, P = 0.002), compared to human bronchial epithelial cells (all P < 0.05, data not shown).

### The effect of HOXA11-AS on proliferation, migration, invasion, apoptosis and cell cycle in NSCLC *in vitro*

**Figure 3:**
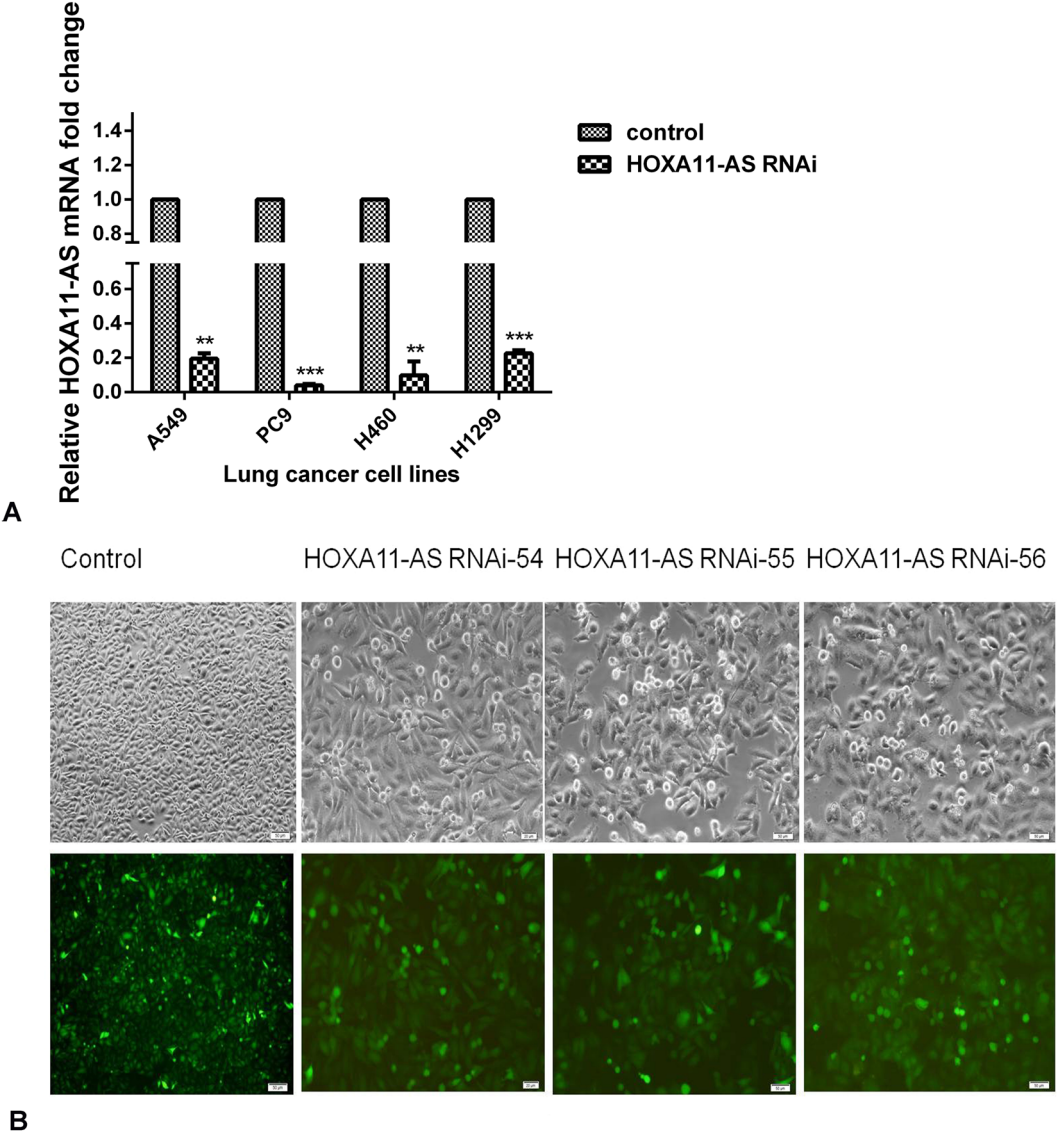
The expression of HOXA11-AS in different groups. (**A**) The relative expression of HOXA11-AS were detected in NSCLC cells (A549, PC9, H460 and H1299) after transfection with lenti-control virus and HOXA11-AS RNAi (Note: *P < 0.05, **P < 0.01, ***P < 0.001, control: lenti-control virus). (**B**) Light microscope and fluorescence microscope images of A549 cells after transfection with lenti-control virus and HOXA11-AS RNAi (Control 40× and HOXA11-AS RNAi 200×).

To explore the underlying effect of HOXA11-AS on biological processes in NSCLC, lentivirus-mediated HOXA11-AS RNAi was constructed. Thus, we observed the transfection efficiency of HOXA11-AS RNAi under a light microscope and fluorescence microscope. We found that the transfection efficiency of HOXA11-AS RNAi group in NSCLC cell lines was over 80%, and the knockdown efficiency of HOXA11-AS was over 75%. Furthermore, real time RT-qPCR was used to detect the expression of HOXA11-AS in different groups, and we found that HOXA11-AS expression was significantly lower in HOXA11-AS RNAi groups than in control groups (P < 0.01, Fig. 3A). In A549 cells, the expression of HOXA11-AS was remarkably reduced after transfection with HOXA11-AS RNAi (t = 25.53, P = 0.002). The light microscope and fluorescence microscope images after transfection with lenti-control virus and HOXA11-AS RNAi are shown in Fig. 3B. Similar to A549 cells, a significant decrease after transfection with HOXA11-AS RNAi was observed in the other 3 cell lines (PC9, H460 and H1299) (data not shown).

**Figure 4:**
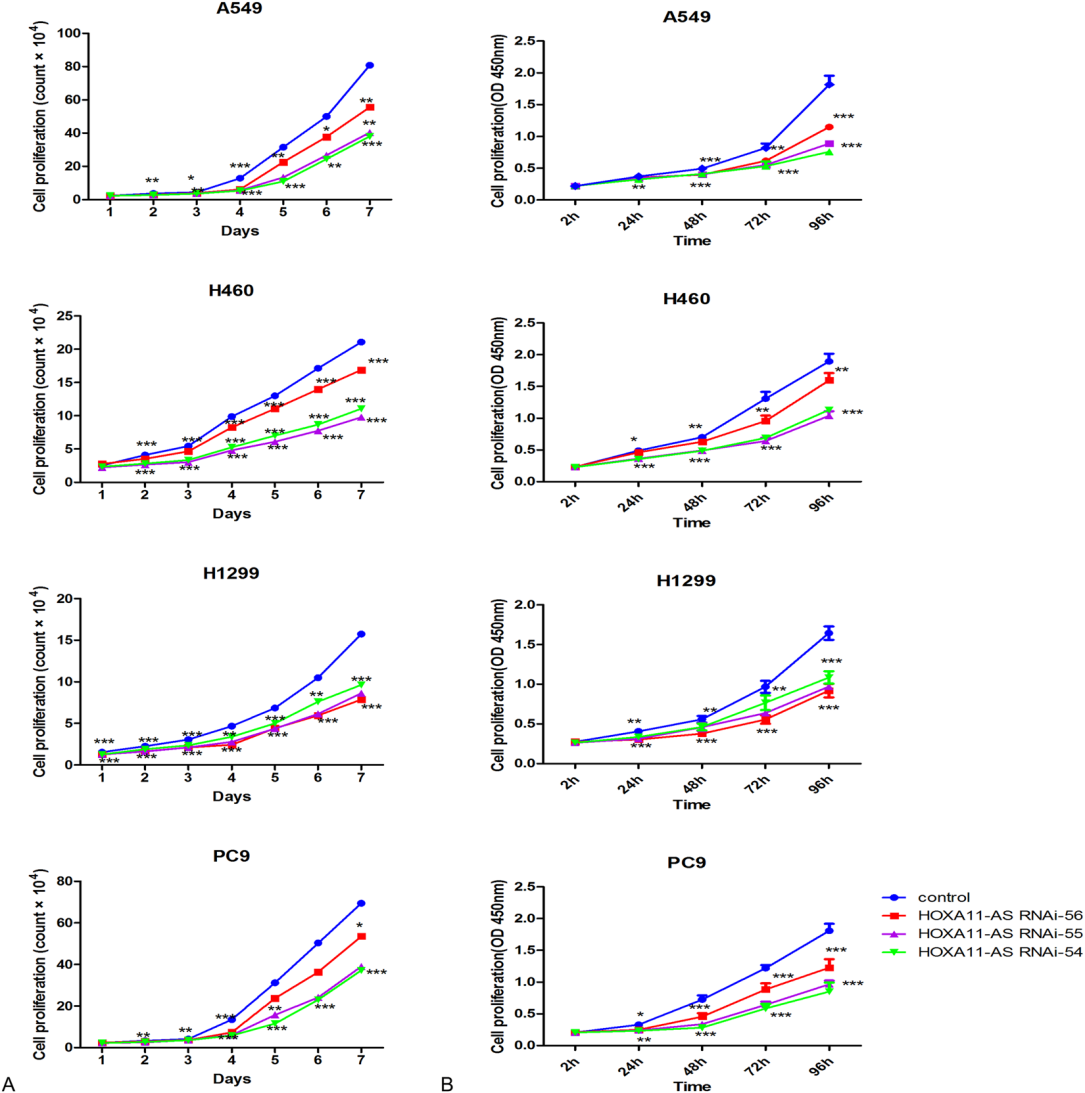
HOXA11-AS RNAi inhibited the proliferation of A549, H460, H1299 and PC9 cell lines (*P < 0.05, **P < 0.01, ***P < 0.001).(Note: control, lenti-control virus) The proliferation of A549, H460, H1299 and PC9 cell lines was inhibited based on a cytometry assay (**A**) and CCK-8 assay (**B**).

To further evaluate the proliferation of cells in 3 different groups (blank control, lenti-control virus group and HOXA11-AS RNAi group), cytometry and CCK-8 assays were performed. We observed a significant inhibition in proliferation in the HOXA11-AS RNAi group of all (A549, H460, 1299 and PC9) cell lines compared to control groups (Fig. 4). In the cytometry assay, the proliferation of the HOXA11-AS RNAi group was clearly suppressed (P < 0.05), although a minor difference was observed between the different HOXA11-AS RNAi groups (HOXA11-AS RNAi-54, HOXA11-AS RNAi-55, HOXA11-AS RNAi-56). A more obvious suppression of proliferation was found in HOXA11-AS RNAi-54 and HOXA11-AS RNAi-55 groups than in HOXA11-AS RNAi-56 groups in A549, H460 and PC9 cell lines, whereas proliferation of H1299 cells was clearly found to be inhibited in HOXA11-AS RNAi-56 groups. Specifically, in A549 cells, no obvious difference was found between HOXA11-AS RNAi-54 and HOXA11-AS RNAi-55 groups compared with control groups on the first day. Whereas, on the second day, the proliferation of HOXA11-AS RNAi-54 and HOXA11-AS RNAi-55 groups was remarkably reduced (P = 0.002). Then, on days 3 to 7, the proliferation was also obviously reduced in HOXA11-AS RNAi-54 and HOXA11-AS RNAi-55 groups (P < 0.01). In the HOXA11-AS RNAi-56 group, no obvious difference was found on the first day (P = 0.591), whereas a significant decrease was found on days 2 to 7 (P < 0.05, Table 1). Comparable results were achieved in the other 2 cell lines (PC9 and H460, data not shown). For H1299 cells, the strongest inhibition was found in the HOXA11-AS RNAi-56 group (P < 0.01, data not shown). To verify the results of the cytometry assay, a cell counting kit-8 (CCK-8) assay was performed, and similar conclusions were demonstrated by the CCK-8 assay in A549 cells (Table 2). Knockdown of HOXA11-AS inhibited the proliferation of the 4 NSCLC cell lines. The HOXA11-AS RNAi-54 groups exhibited obvious proliferation suppression in A549, H460 and PC9 cell lines, consistent with the HOXA11-AS RNAi-55 group (P < 0.001). However, the HOXA11-AS RNAi-56 group showed the most significant inhibition in H1299 cells (P < 0.001, data not shown). Based on these results, we can conclude that HOXA11-AS markedly increases the proliferation of these 4 NSCLC cell lines.

**Table 1:** Effect of HOXA11-AS RNAi on proliferation of the NSCLC A549 cell line, determined by cytometry assays.

Time	Control	HOXA11-AS RNAi-54			HOXA11-AS RNAi-55			HOXA11-AS RNAi-56		
	Mean ± SD	Mean ± SD	t	*p*	Mean ± SD	t	*p*	Mean ± SD	t	*p*
Day 1	2.293 ± 0.040	2.273 ± 0.042	0.618	0.57	2.278 ± 0.040	0.477	0.658	2.274 ± 0.041	0.583	0.591
Day 2	3.724 ± 0.194	2.790 ± 0.121	7.075	0.002	2.775 ± 0.105	7.442	0.002	2.842 ± 0.125	6.615	0.003
Day 3	4.448 ± 0.125	3.571 ± 0.203	6.347	0.003	3.681 ± 0.204	5.547	0.005	3.870 ± 0.234	3.768	0.02
Day 4	12.913 ± 0.142	5.385 ± 0.329	36.411	< 0.001	0.5.572 ± 0.364	32.598	< 0.001	6.133 ± 0.310	34.463	< 0.001
Day 5	31.530 ± 0.680	10.966 ± 1.056	28.361	< 0.001	13.336 ± 0.629	34.025	< 0.001	22.653 ± 2.140	6.848	0.002
Day 6	49.923 ± 4.801	24.352 ± 2.199	8.386	< 0.001	26.706 ± 2.007	7.727	0.002	37.730 ± 3.272	3.635	0.022
Day 7	80.793 ± 6.610	38.057 ± 2.358	10.547	< 0.001	40.394 ± 1.736	10.238	0.001	55.600 ± 5.568	5.049	0.007

t values were obtained by comparison with the negative control.

**Table 2:** Effect of HOXA11-AS RNAi on proliferation of NSCLC A549 cell line, determined by CCK-8 assay.

time	Control	HOXA11-AS RNAi-54			HOXA11-AS RNAi-55			HOXA11-AS RNAi-56		
	Mean ± SD	Mean ± SD	t	*p*	Mean ± SD	t	*p*	Mean ± SD	t	*p*
2 h	0.219 ± 0.001	0.221 ± 0.003	−1.342	0.251	0.221 ± 0.002	−1.414	0.230	0.222 ± 0.015	−2.475	0.069
24 h	0.369 ± 0.158	0.324 ± 0.018	4.143	0.003	0.346 ± 0.016	2.335	0.048	0.353 ± 0.007	2.087	0.07
48 h	0.494 ± 0.021	0.411 ± 0.033	4.732	0.001	0.402 ± 0.009	8.875	< 0.001	0.397 ± 0.013	8.527	< 0.001
72 h	0.818 ± 0.068	0.531 ± 0.074	6.334	< 0.001	0.552 ± 0.018	8.395	0.001	0.616 ± 0.004	6.582	0.003
96 h	1.816 ± 0.138	0.757 ± 0.031	16.738	< 0.001	0.886 ± 0.011	15.022	< 0.001	1.147 ± 0.022	10.692	< 0.001

t values were obtained by comparison with the negative control.

**Figure 5:**
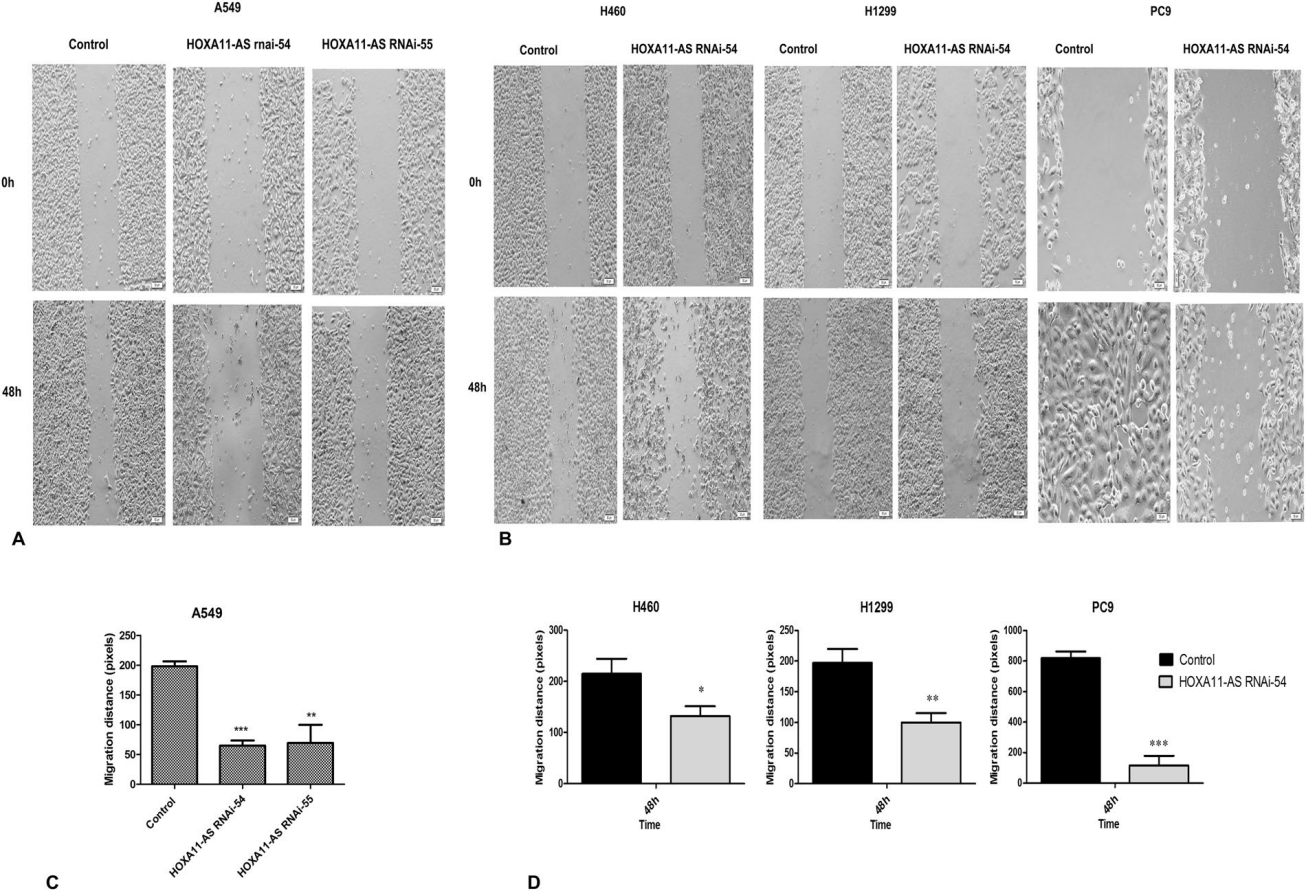
HOXA11-AS RNAi inhibited the migration of A549, H460, H1299 and PC9 cell lines, based on a cell scratch assay. (Note: control, lenti-control virus). (**A**) HOXA11-AS RNAi inhibited cell migration ability in A549 cells. (**B**) HOXA11-AS RNAi inhibited cell migration ability in H460, H1299 and PC9 cell lines. (**C**) The migration distance of control, HOXA11-AS RNAi-54 and HOXA11-AS RNAi-55 groups in A549 cells at 0 h and 4 8 h (40×) (*P < 0.05, **P < 0.01, ***P < 0.001). (**D**) The migration distance of control and the HOXA11-AS RNAi-54 group in H460, H1299 and PC9 cell lines at 0 h and 48 h (40×) (*P < 0.05, **P < 0.01, ***P < 0.001).

**Figure 6:**
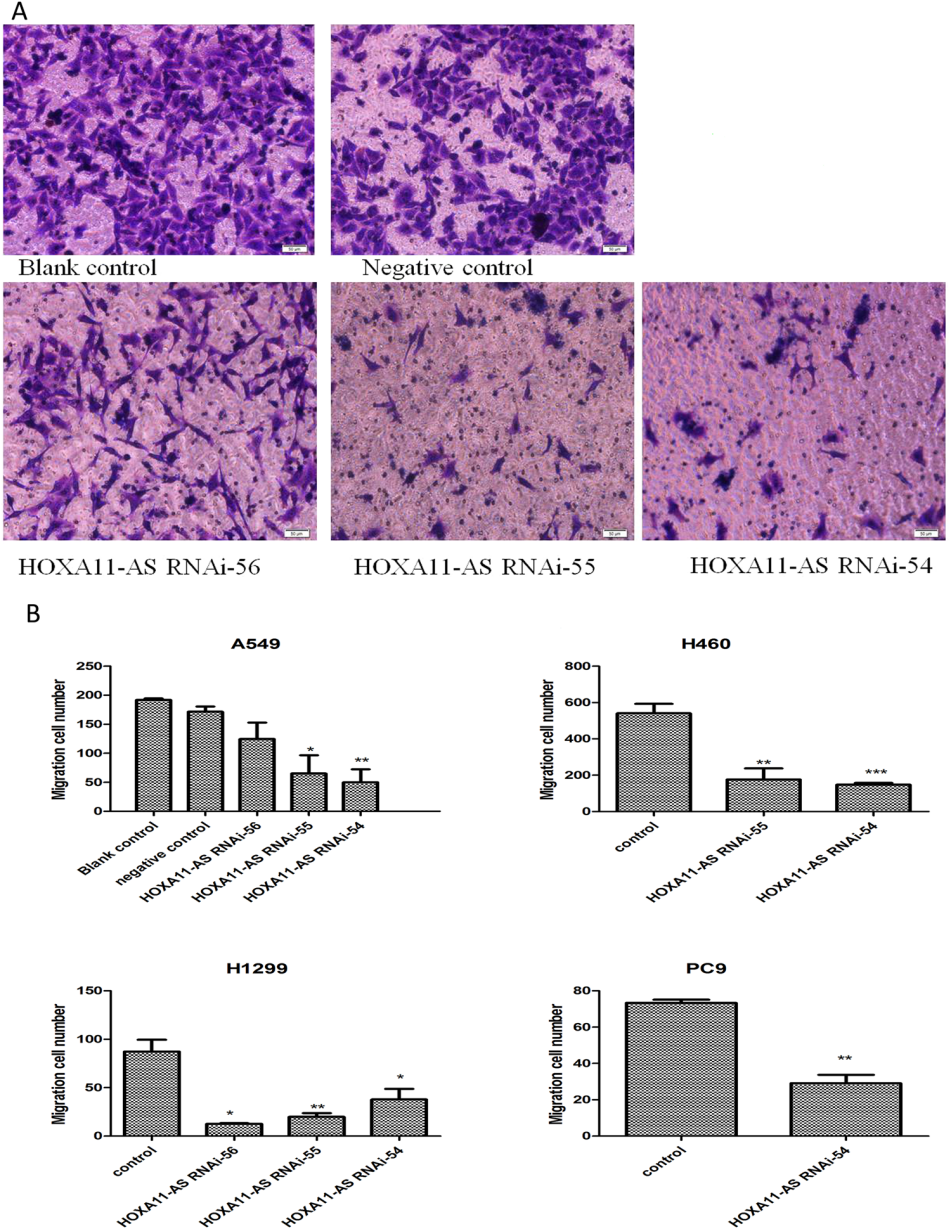
HOXA11-AS RNAi inhibited the migration of A549, H460, H1299 and PC9 cell lines, based on a Transwell migration assay. (**A**) Transwell migration assays were performed to detect the migration ability of A549 cells after transfection with HOXA11-AS RNAi. A549 cells were transfected with the blank control, lenti-control virus and three different interference sequences of HOXA11-AS RNAi (HOXA11-AS RNAi-54, HOXA11-AS RNAi-55 and HOXA11-AS RNAi-56). After 24 hours, cells were stained with crystal violet, and the migratory cells were observed with a microscope (200×). (**B**) The numbers of migratory cell in different cell lines.

Then, a cell scratch assay was performed to further explore the effect of HOXA11-AS RNAi on the migration ability of NSCLC cells. The results demonstrated that the migration ability was significantly inhibited after knockdown of HOXA11-AS, especially in A549 cells (P < 0.01, Fig. 5A). In addition, among the 3 HOXA11-AS RNAi groups, the HOXA11-AS RNAi-54 group was found to have the strongest ability to suppress migration at 48 h (P < 0.001, Fig. 5C). Similar results were obtained in H460, H1299 and PC9 cell lines (P < 0.05, Fig. 5B,D). Overall, these results indicated that the migration of NSCLC cells was inhibited due to knockdown of HOXA11-AS. Furthermore, a Transwell migration assay was used to further verify the results of the cell scratch assay. Consistent with the cell scratch assay, the migration ability in the HOXA11-AS RNAi-54 group was remarkably suppressed in the A549 cell line compared to the control group (P = 0.007, Fig. 6A). A similar inhibition of migration was found in H460 and PC9 cells (P < 0.01), whereas in H1299 cells, the HOXA11-AS RNAi-56 group demonstrated the strongest inhibition effect (P = 0.026, Fig. 6B). We can conclude that HOXA11-AS significantly promotes the migration of NSCLC cells.

**Figure 7:**
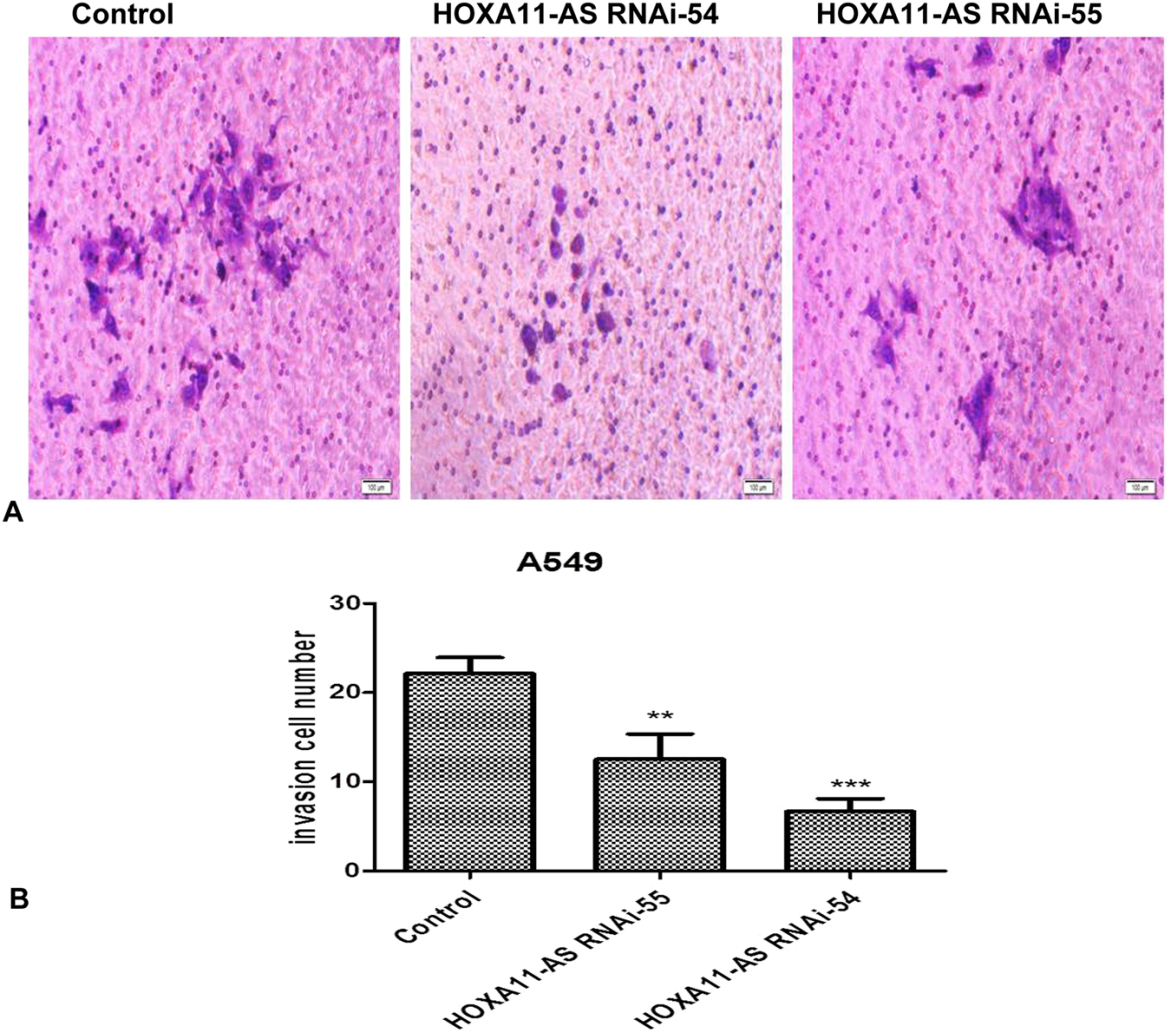
HOXA11-AS RNAi inhibited the invasion ability of A549 cells. (Note: control, lenti-control virus. (**A**) Qualitative Transwell migration assay of A549 cell invasion after knocking down HOXA11-AS. (**B**) HOXA11-AS RNAi-54 markedly inhibited the invasion ability of the A549 cells. (**P < 0.01, ***P < 0.001).

Furthermore, the effect of HOXA11-AS RNAi on invasion ability was detected in NSCLC cells using a Transwell invasion assay. In A549 cells, we demonstrated that HOXA11-AS RNAi could reduce cell invasion ability, compared to control groups (P < 0.001, Fig. 7A). Additionally, a more remarkable inhibition was found in the HOXA11-AS RNAi-54 group than in the HOXA11-AS RNAi-55 group (P = 0.033, Fig. 7B). Similar results were found in H460, H1299 and PC9 cell lines (data not shown).

**Figure 8:**
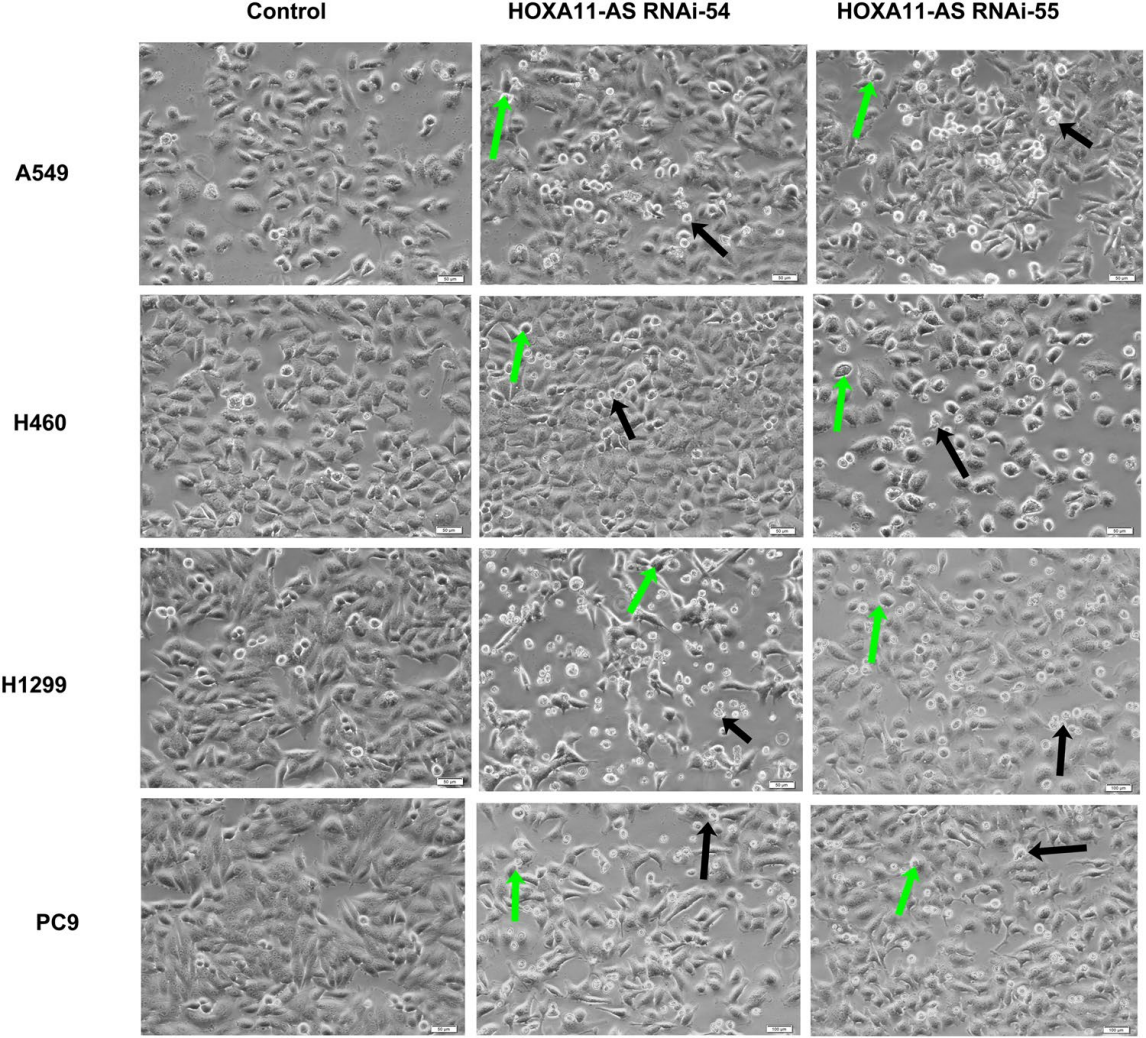
HOXA11-AS RNAi promoted apoptosis in A549, H460, H1299 and PC9 cell lines. Four NSCLC cell lines (A549, H460, H1299 and PC9) were transfected separately with lenti-control virus, HOXA11-AS RNAi-54 and HOXA11-AS RNAi-55. An increased number of apoptotic cells (green arrows) and dead cells (black arrows) were observed under a light microscope (200×).

**Figure 9:**
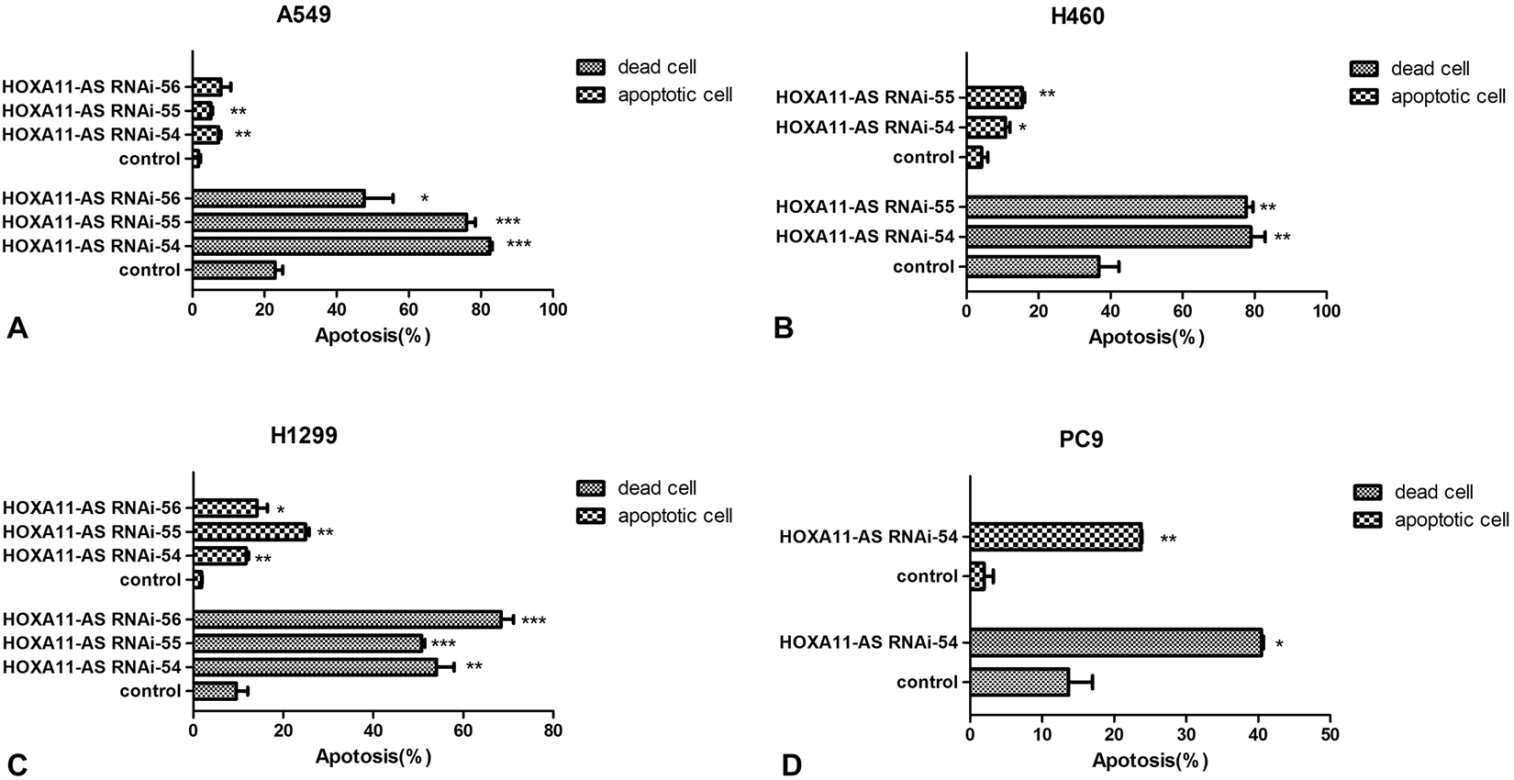
HOXA11-AS RNAi promoted apoptosis in A549, H1299, H460 and PC9 cell lines based on a flow cytometry assay. (Note: control, lenti-control virus). (**A**) Comparison of the apoptosis rate and mortality rate of A549 cells after silencing HOXA11-AS expression (*P < 0.05, **P < 0.01, ***P < 0.001). (**B**) Comparison of the apoptosis rate and mortality rate of H460 cells after silencing HOXA11-AS expression (*P < 0.05, **P < 0.01, ***P < 0.001). (**C**) Comparison of the apoptosis rate and mortality rate of H1299 cells after silencing HOXA11-AS expression (*P < 0.05, **P < 0.01, ***P < 0.001). (**D**) Comparison of the apoptosis rate and mortality rate of PC9 cells after silencing HOXA11-AS expression (*P < 0.05, **P < 0.01, ***P < 0.001).

Afterwards, we focused on the effect of HOXA11-AS on cell apoptosis in these 4 NSCLC cell lines (A549, H460, H1299 and PC9) via a flow cytometry assay. After being transfected with HOXA11-AS RNAi, an increased number of apoptotic cells and dead cells could be observed under a light microscope, indicating that HOXA11-AS RNAi could significantly promote apoptosis in NSCLC cells (Fig. 8). To further verify the acceleration effect of HOXA11-AS RNAi on apoptosis, Annexin V-PE/7-AAD staining was performed to measure cell apoptosis in these 4 NSCLC cell lines. In A549 cells, we found that the cell apoptotic rate increased in the groups that were transfected with HOXA11-AS RNAi-54 (7.26 ± 1.94%) and HOXA11-AS RNAi-55 (5.15 ± 0.74%) compared to those in the lenti-control virus group (1.72 ± 0.94%, all P < 0.01, Fig. 9A). Consistent results were obtained in H460, H1299 and PC9 cell lines, indicating that HOXA11-AS could clearly inhibit apoptosis in these 4 NSCLC cell lines (Fig. 9B–D).

**Figure 10:**
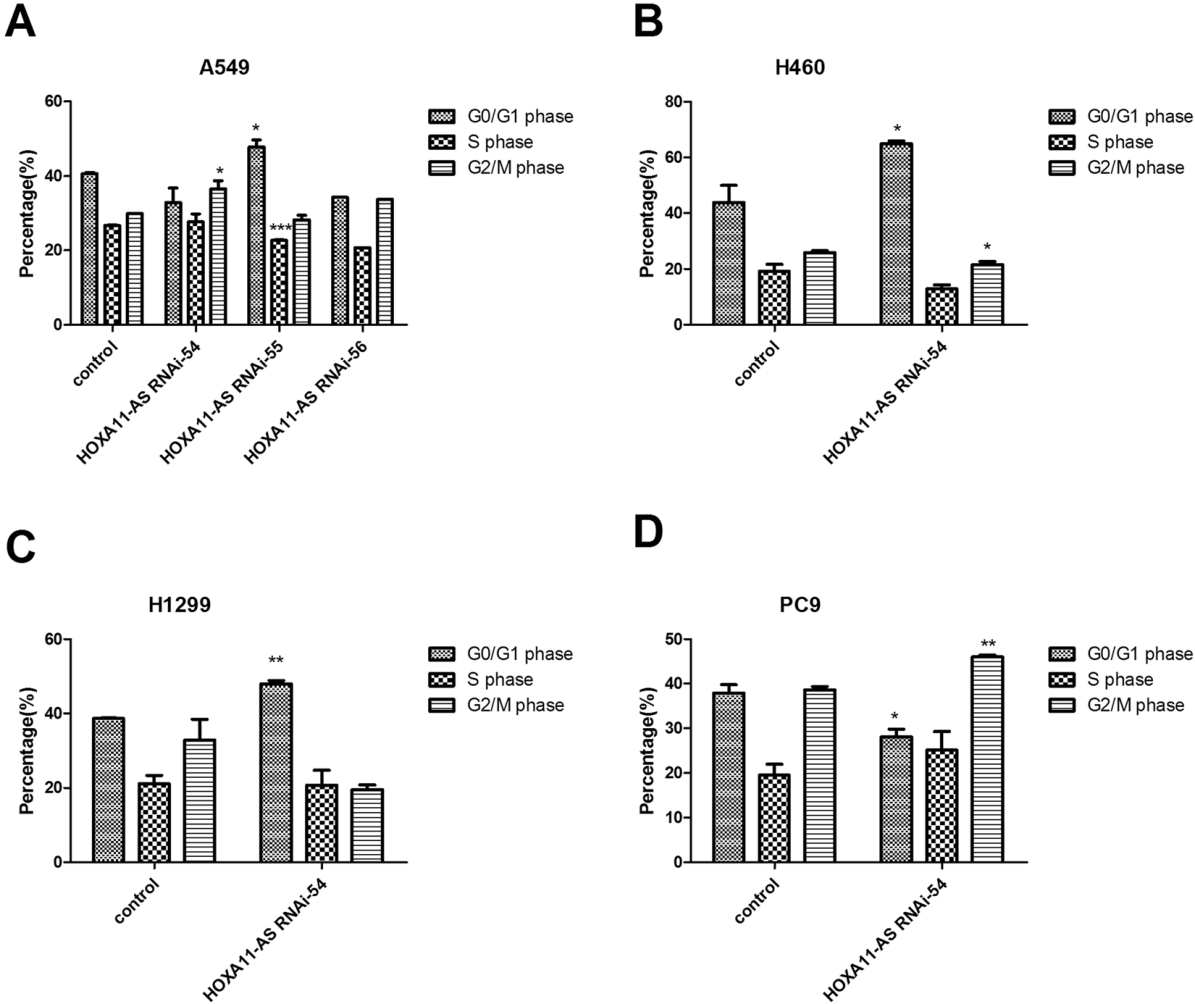
The cell cycle distribution of A549, H460, H1299 and PC9 cells after silencing HOXA11-AS expression. (Note: control, lenti-control virus) (**A**) A549 cells, (**B**) H460 cells, (**C**) H1299 cells, (**D**) PC9 cells.

Additionally, flow cytometry was used to measure the effect of HOXA11-AS on cell cycle distribution. In A549 cells, the results revealed that HOXA11-AS RNAi-54 caused cell cycle arrest on G2/M phase, whereas HOXA11-AS RNAi-55 caused cell cycle arrest G0/G1 phase compared with the lenti-control virus group (all P < 0.05). No significant fluctuation was found in S phase among the 3 groups (HOXA11-AS RNAi-54, HOXA11-AS RNAi-55, HOXA11-AS RNAi-56, Fig. 10A). Furthermore, in H460 and H1299 cells, HOXA11-AS RNAi-54 caused cell cycle arrest on G0/G1 phase, whereas in PC9 cells, a clear cycle arrest on G2/M phase in the HOXA11-AS RNAi-54 group was reduced (Fig. 10B–D). Considering the motivating effect of HOXA11-AS on proliferation, a rational inference was made that HOXA11-AS could improve the proliferation of NSCLC, in part because HOXA11-AS activated the cell cycle process.

### HOXA11-AS promotes NSCLC tumorigenesis and angiogenesis *in vivo*

**Figure 11:**
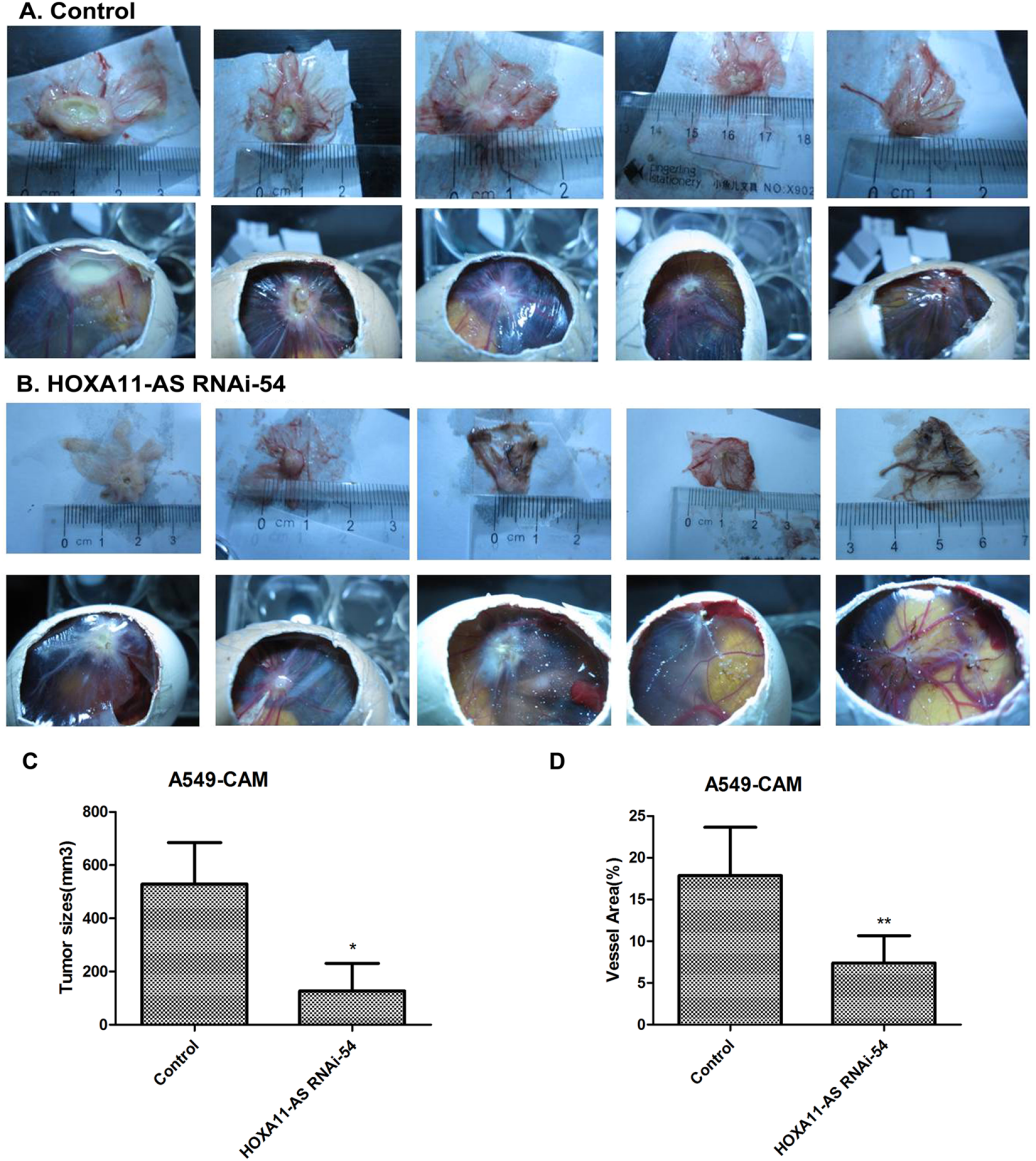
The A549 cell tumor formation in the chick chorioallantoic membrane after transfecting A549 cells with HOXA11-AS RNAi. (**A**) Lenti-control virus group. (**B**) HOXA11-AS RNAi-54 group. (**C**) Comparison of the size of the A549 cell tumor xenografts after silencing HOXA11-AS expression (*P < 0.05). (**D**) Comparison of the vascular area ratio of the A549 cell tumor xenografts after silencing HOXA11-AS expression (**P < 0.01).

**Figure 12:**
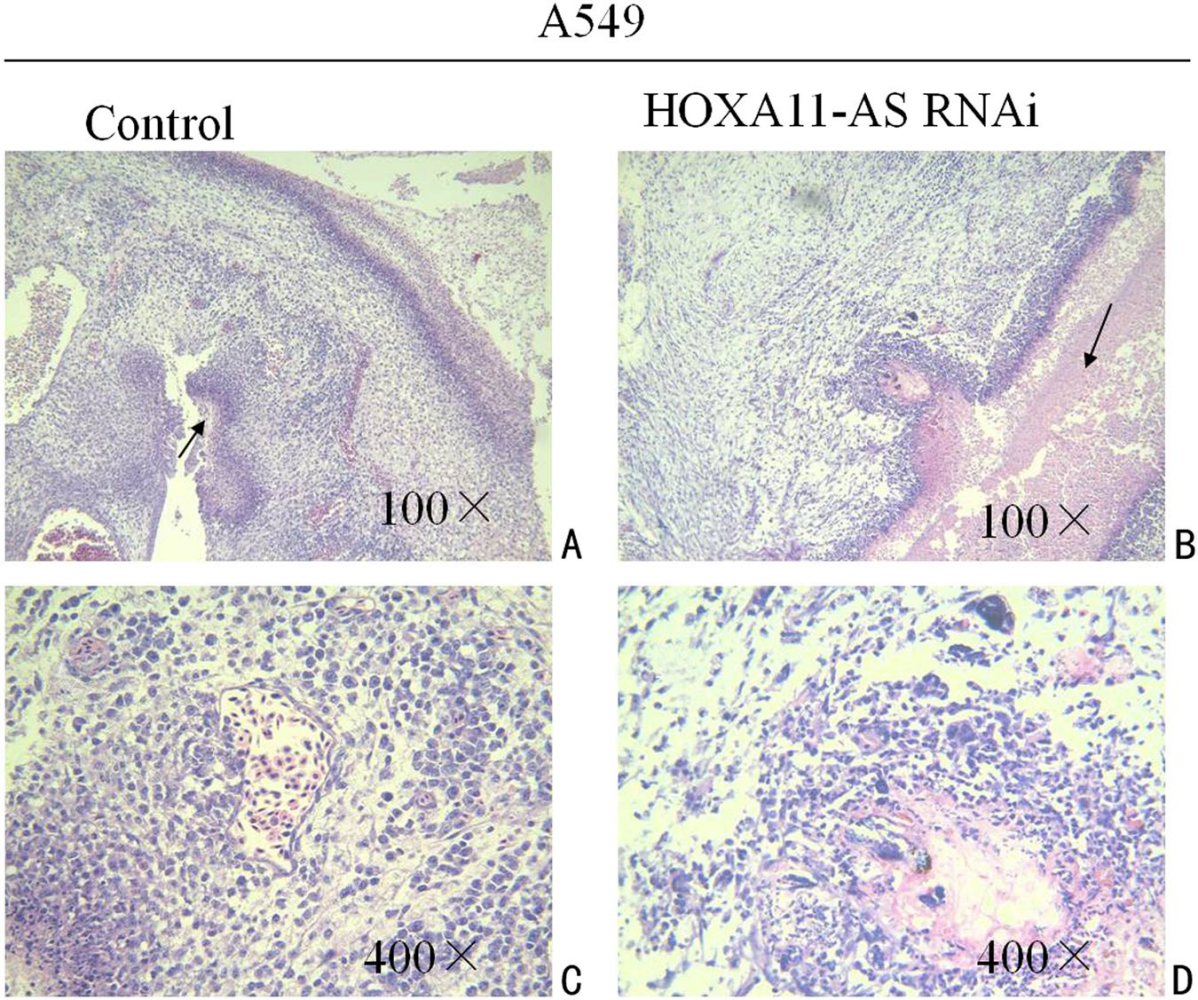
Hematoxylin/eosin (HE) staining of the tumor xenografts. (**A**) Hematoxylin/eosin (HE) staining of the tumor xenografts in the lenti-control virus group (100×). (**B**) Hematoxylin/eosin (HE) staining of the tumor xenografts in the HOXA11-AS RNAi group (100×). (**C**) Hematoxylin/eosin (HE) staining of the tumor xenografts in the lenti-control virus group (400×). (**D**) Hematoxylin/eosin (HE) staining of the tumor xenografts in the HOXA11-AS RNAi group (400×).

Tumor formation experiments in a CAM model with A549, H460, H1299 and PC9 cells were conducted to assess the effect of HOXA11-AS on tumorigenesis and angiogenesis *in vivo*. The results showed that both the lenti-control virus group and HOXA11-AS RNAi group could form tumor xenografts, and the tumorigenic and angiogenic ability of all 4 cell lines was weakened by HOXA11-AS RNAi (P < 0.05). In A549 cells, the size of the tumor xenografts was significantly reduced after transfection with HOXA11-AS RNAi-54 (127.1 ± 232.728 mm^3^ vs 528.667 ± 382.206 mm^3^, t = −2.008, P = 0.045) compared with the lenti-control virus group. In addition to the effect on tumor size, the HOXA11-AS RNAi-54 group also exhibited a decreased vascular area ratio (7.396 ± 3.266) of tumor formation in chick chorioallantoic membrane compared to the lenti-control virus group (17.867 ± 5.795, t = 3.575, P = 0.006, Fig. 11). Furthermore, based on hematoxylin/eosin (HE) staining, we found that the original morphology of NSCLC cells was maintained in tumor xenografts. In addition, inflammatory cells and noticeable necrosis were found in pathological sections of the tumor xenografts (Fig. 12).

### The potential pathways associated with HOXA11-AS

**Figure 13:**
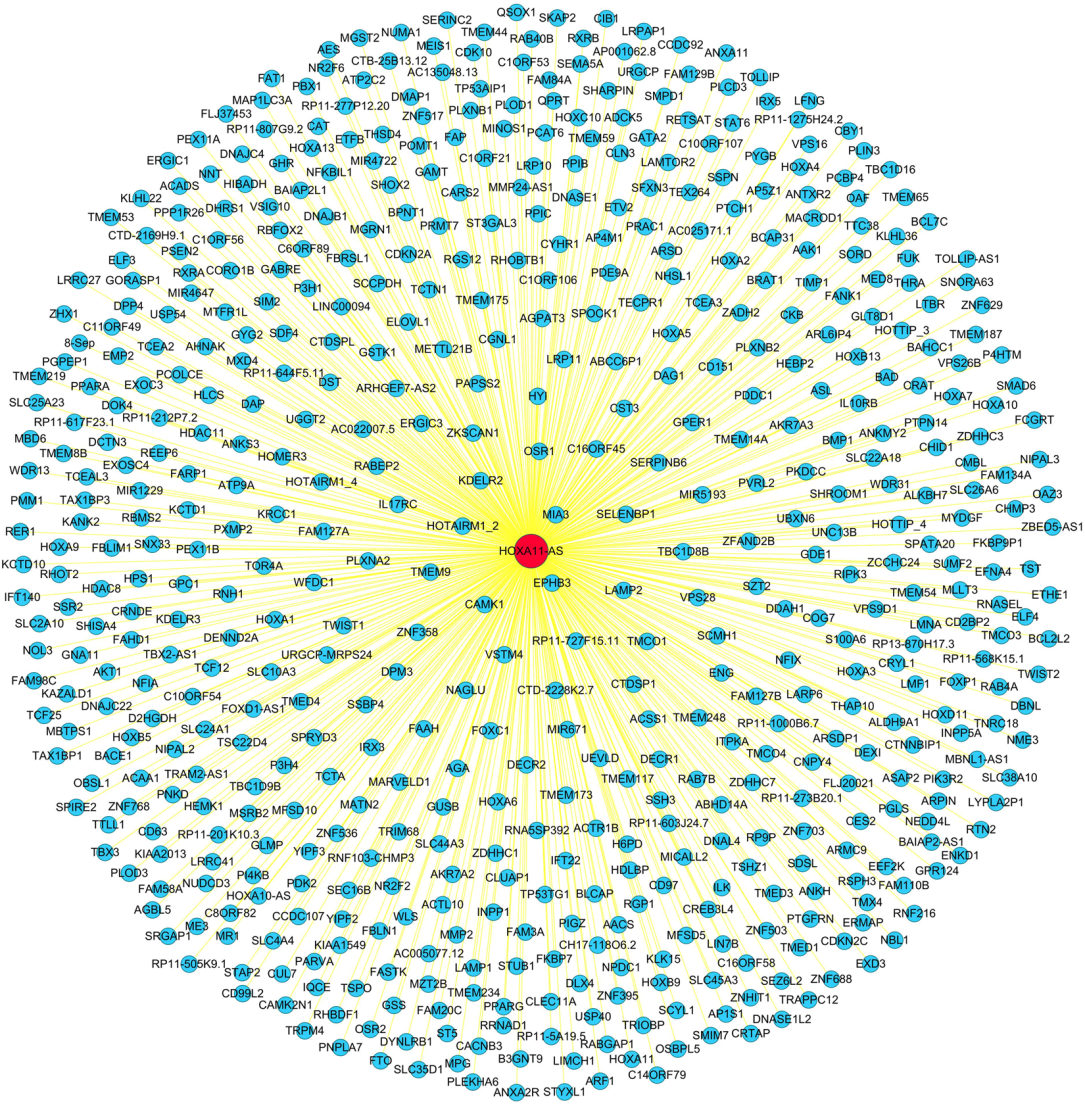
The network of 574 co-expressed genes of HOXA11-AS overlapping in two probe sets (230666_AT and 239950_AT).

**Figure 14:**
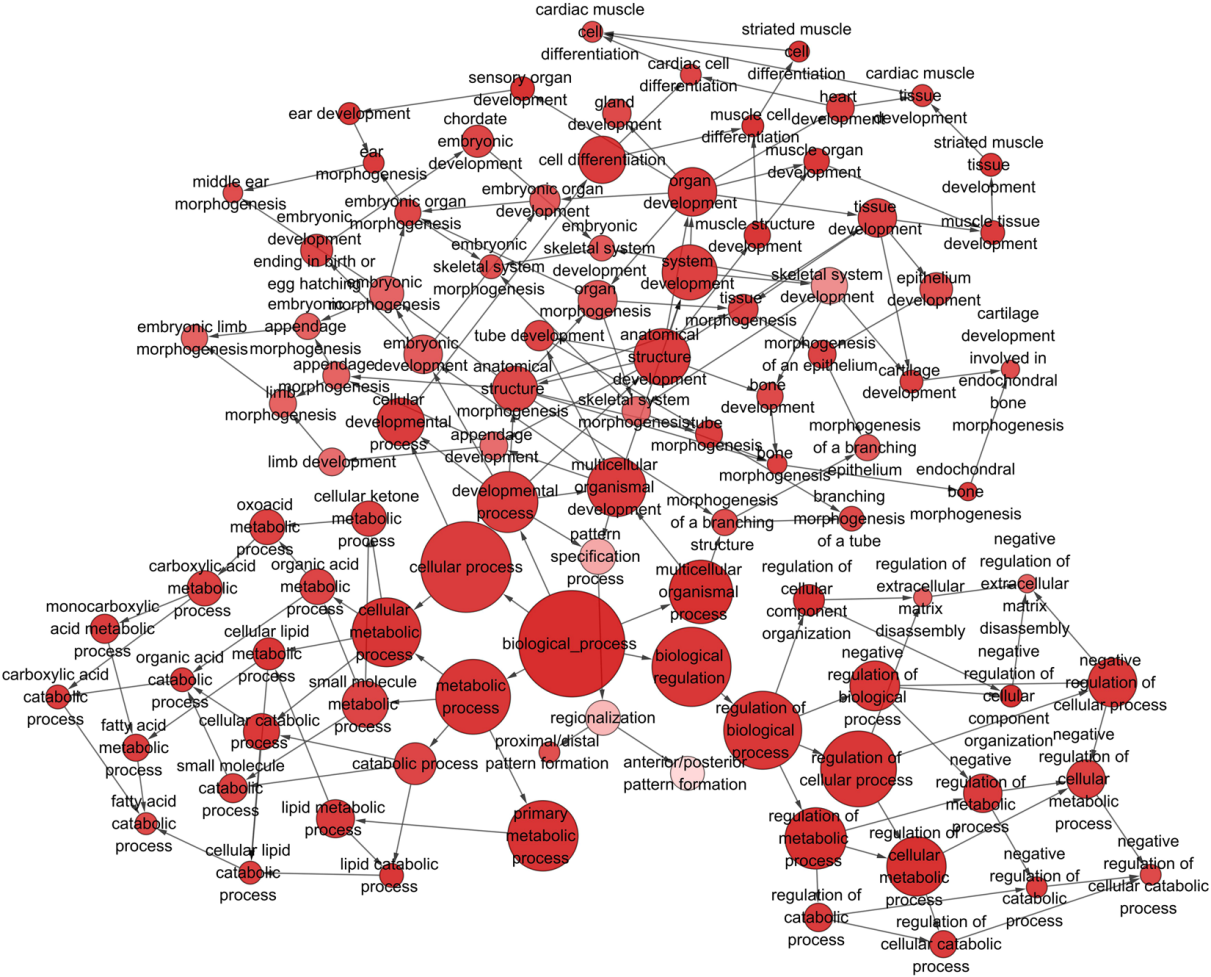
A function network of Gene Ontology (GO) terms for the co-expressed genes of HOXA11-AS in NSCLC.

An *in silico* analysis was performed to explore HOXA11-AS-associated biological pathways based on MEM^21, 22^ and the DAVID Bioinformatics Tool^23, 24^. We collected the top 1500 co-expressed genes in different probe sets for HOXA11-AS (230666_AT and 239950_AT) as previously reported^25, 26^. Then, the 574 correlated genes overlapping in these two probe sets were selected for the downstream analysis (Fig. 13). Furthermore, we explored gene potential functional enrichment by Gene ontology (GO) analysis based on these 574 HOXA11-AS co-expressed genes. Then, the significant enriched biological terms were identified by the threshold of P-value less than 0.05. Consequently, positive regulation of transcription from RNA polymerase was revealed to be most strongly enriched biological term. Nobly, the result also showed that regulation of cell migration, as well as extracellular space and protein binding were strongly enriched biological term, which were closely related to the progress of cancer. To better understand the functions of these co-expressed genes, a function network was constructed based on the GO analysis (Fig. 14).

**Figure 15:**
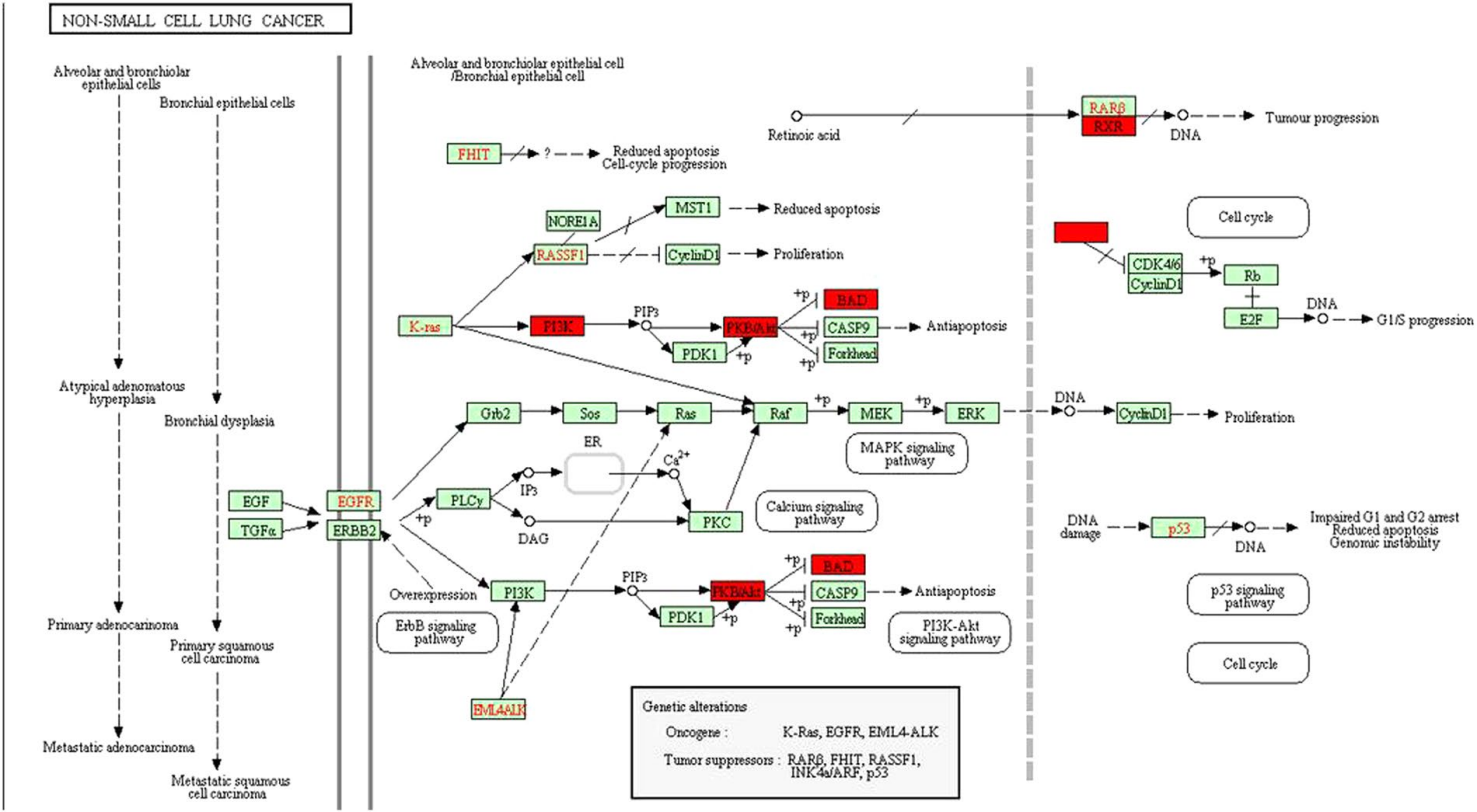
HOXA11-AS co-expressed genes were significantly overrepresented in the non-small cell lung cancer pathway, revealed by KEGG pathway analysis^52–54^ (http://www.kegg.jp/kegg/kegg1.html).

In addition, the Kyoto encyclopedia of genes and genomes (KEGG) pathway analysis revealed that the HOXA11-AS co-expressed genes were significantly overrepresented in the non-small cell lung cancer pathway, supporting our aforementioned result that HOXA11-AS might play a vital role in NSCLC (Fig. 15). The top five most significant GO terms and the top ten KEGG pathway items are presented in Table 3 and Table 4. Altogether, the GO terms and KEGG pathway items reinforced the observation that HOXA11-AS might be involved in biological mechanisms in NSCLC.

**Table 3:** The top 5 enrichment GO terms (BP, CC, and MF) of the co-expressed genes of HOXA11-AS.

GO ID	GO term	Count	*P* value
**Biological process**			
GO:0006874	cellular calcium ion homeostasis	12	4.37E-06
GO:0050731	positive regulation of peptidyl-tyrosine phosphorylation	11	1.05E-05
GO:0045944	positive regulation of transcription from RNA polymerase	42	1.43E-05
GO:0006955	immune response	24	2.09E-05
GO:0030334	regulation of cell migration	10	3.09E-05
**Cellular component**			
GO:0005615	extracellular space	53	1.61E-06
GO:0043234	protein complex	21	1.63E-04
GO:0016020	membrane	67	1.64E-04
GO:0005829	cytosol	94	1.69E-04
GO:0005913	cell-cell adherens junction	18	1.90E-04
**Molecular function**			
GO:0005515	protein binding	182	3.61E-09
GO:0019904	protein domain specific binding	17	6.84E-06
GO:0043565	sequence-specific DNA binding	25	2.38E-04
GO:0098641	cadherin binding involved in cell-cell adhesion	16	0.001168
GO:0032403	protein complex binding	13	0.001428

Note: GO, Gene Ontology.

**Table 4:** KEGG pathway enrichment analysis of the co-expressed genes of HOXA11-AS.

KEGG ID	KEGG term	Count	*P* value
hsa00920	Sulfur metabolism	4	0.000273
hsa04142	Lysosome	10	0.001434
hsa04146	Peroxisome	8	0.001572
hsa05223	Non-small cell lung cancer	6	0.003697
hsa04360	Axon guidance	11	0.005748
hsa05202	Transcriptional misregulation in cancer	11	0.006707
hsa00280	Valine, leucine and isoleucine degradation	5	0.008757
hsa00514	Other types of O-glycan biosynthesis	4	0.009583
hsa03320	PPAR signaling pathway	6	0.011214
hsa01522	Endocrine resistance	7	0.012822

Notes: KEGG, Kyoto Encyclopedia of Genes and Genomes.

## Discussion

In this study, TCGA database was used to explore the differential expression of HOXA11-AS between normal lung and NSCLC tissues. QRT-PCR and flow cytometry as well as CCK-8, wound healing, migration, Matrigel invasion, and cytometry assays, were performed to determine the effect of HOXA11-AS in NSCLC cell lines. Moreover, a CAM model of NSCLC was constructed to explore the effect of HOXA11-AS on tumorigenicity and angiogenesis in NSCLC. In addition, MEM was used to further analyze the co-expressed genes and potential pathways associated with HOXA11-AS. As a result, our data confirms that HOXA11-AS was up-regulated in both lung adenocarcinoma and squamous cell carcinoma compared to normal lung tissues based on TCGA database. Additionally, we found that the AUC of HOXA11-AS was 0.878 (95% CI: 0.786–0.969) for lung adenocarcinoma and 0.962 (95% CI: 0.928–0.996) for lung squamous cell carcinoma, which predicted a diagnostic value of HOXA11-AS in NSCLC. Consistent with the results from TCGA, qRT-PCR showed an up-regulated trend in the HOXA11-AS level in NSCLC tissues compared to corresponding non-cancerous lung tissues. To further explore the biological role of HOXA11-AS expression *in vitro* and *in vivo*, we found that HOXA11-AS was overexpressed in all of the 4 NSCLC cell lines (A549, H460, H1299 and PC9). Then, we performed various experiments to confirm that HOXA11-AS was a tumorigenic gene. The proliferation, migration, invasion and tumorigenic and angiogenic ability of NSCLC cells were all inhibited and apoptosis was induced after silencing the expression of HOXA11-AS. Migration and invasion is the prerequisite for tumor metastasis. Our results indicate that HOXA11-AS might be associated with metastasis in NSCLC. After examining apoptosis and cell cycle distribution by flow cytometry, a rational inference was made that HOXA11-AS RNAi could suppress the proliferation of NSCLC in part because HOXA11-AS RNAi caused cell cycle arrest on different phases. Phases such as G1/G0, S, and G2/M represent the different steps during cell proliferation^27^. In our study, HOXA11-AS knockdown causes G0/G1 or G2/M arrest and cell cycle could not progress to the following phase, which indicate that the silencing of HOXA11-AS inhibit cell cycle progression, also, the different phenotypes of HOXA11-AS RNAi may cause a minor difference on different phases arrest. In addition, as reported, deregulated cell-cycle control is a fundamental aspect for the treatment of malignancies^28^. Up to now, the detailed molecular mechanisms by which HOXA11-AS affect cell cycle remain elusive. Cyclin, p53, p21, p27 and transcription factor Sp1 were all involved in different phases of the cycle as reported^27, 29, 30^. The difference expression of these cell-cycle regulation proteins over time might be associated with cell-cycle arrest and apoptotic cell death^27, 30^. However, further studies are needed to fully identify the mechanism how HOXA11-AS regulate cell cycle. In addition, angiogenesis is the basis of tumor growth, metastasis, invasion and recidivism, and the results of the CAM model confirmed that the tumorigenic and angiogenic ability of NSCLC cells was weakened after silencing the expression of HOXA11-AS, indicating that HOXA11-AS has a promoting effect on angiogenesis. These results suggest that HOXA11-AS can act as an oncogene to accelerate malignant progression and metastasis of NSCLC. In addition, GO analysis of the co-expressed genes revealed that they were involved in complex cellular pathways, such as regulation of cell migration, extracellular space and protein binding. KEGG pathway analysis showed that the non-small cell lung cancer pathway might be involved in regulating these co-expressed genes in lung cancer. We also searched Oncomine (https://www.oncomine.org/)^31^ and the Gene Expression Omnibus (GEO; htt://www.ncbi.nlm.nih.gov/geo/) database^32^, but no positive relationship was found.

HOXA11-AS is the antisense RNA of HOXA11 in HOX family genes. Most of the HOX family genes were expressed in normal lung tissues except HOXA11^33^. Several studies have revealed that HOXA11 promoter methylation is related to different tumors, such as endometrial adenocarcinoma, ovarian cancer and glioblastoma^34–36^. Methylation of HOXA11 was significantly associated with poor prognosis in cancer patients^35, 36^. Hypermethylation of HOXA11 was associated with NSCLC progression by effecting cell proliferation or migration^37^. In addition, as reported, no HOXA11-AS expression was found in normal lung tissues, similar to HOXA11 (http://rna.sysu.edu.cn/deepBase/)^19^. In our current study, we found that HOXA11-AS was expressed in both NSCLC tissues and non-cancerous lung tissues, and an up-regulated trend in the HOXA11-AS level in NSCLC tissues was found compared to corresponding normal lung tissues. Additionally, HOXA11-AS was expressed in different NSCLC cell lines (A549, H460, H1299 and PC9). Weak expression of HOXA11-AS was also detected in normal bronchial epithelial cells, which was in contrast to the absence of HOXA11-AS expression in normal lung tissues. The main cause for this might be frequent passages, cellular senescence and the damage caused by pancreatin digestion, which leads to gene mutations in cells. However, a larger sample size is needed to verify our results.

To date, several studies have reported the expression of HOXA11-AS in different types of cancer, such as gastric cancer, ovarian cancer, glioma, colorectal cancer, cervical cancer and NSCLC^20, 38–44^. For example, Sun^39^ *et al*. demonstrated that HOXA11-AS could affect cell growth, migration, invasion and apoptosis of gastric cancer by analyzing microarray data and performing a series of experiments *in vitro* and *in vivo*. Additionally, Sun^39^ *et al*. investigated the potential mechanism of HOXA11-AS in gastric cancer cells, and they found that HOXA11-AS could play an oncogenic role through the EZH2/HOXA11-AS/LSD1 complex or HOXA11-AS/miR-1297/EZH2 crosstalk. Richards^43^ *et al*. found that HOXA11-AS is a tumor-suppressive lncRNA related to epithelial ovarian cancer cell proliferation and survival by exploring genome-wide association study data and performing various functional studies. Richards^43^ *et al*. also revealed that the effects of HOXA11-AS in ovarian cancer could be modified by germline variants. Kim^40^ *et al*. examined the HOXA11-AS expression in cervical cancer and the bio-functional effect of HOXA11-AS *in vitro* and *in vivo*, and they found that HOXA11-AS overexpression enhanced cell proliferation, migration and invasion *in vitro*, whereas *in vivo* xenograft experiments indicated that HOXA11-AS strongly induced tumor growth. Wang^42^ *et al*. applied a high-throughput microarray and gene set enrichment analysis to confirm that HOXA11-AS is associated with cell cycle and could act as a biomarker in progression of glioma. In addition, they found that HOXA11-AS could affect p16, p21 and p27 expression via competitive endogenous RNAs, epigenetic modification, or chromatin modification methods to regulate proliferation and cell cycle of glioma cells. In this study, HOXA11-AS was shown to be an oncogenic lncRNA in NSCLC, but the molecular mechanism of HOXA11-AS in NSCLC remains unclear. GO and KEGG analysis of the co-expressed genes of HOXA11-AS revealed that they were involved in complex cellular pathways, such as regulation of cell migration, extracellular space and protein binding, as well as the non-small cell lung cancer pathway. We hypothesize that HOXA11-AS could affect proliferation, invasion, migration, apoptosis and cell cycle of NSCLC, in part because HOXA11-AS affected the non-small cell lung cancer pathway, such as the ErbB signaling pathway, PI3K-Akt signaling pathway and MAPK signaling pathway. An inappropriate activation of K-Ras, EGFR, RARB and RASSF1 might lead to the development of NSCLC. In addition, our data confirm for the first time the clinical significance and effect of HOXA11-AS on biological processes in NSCLC. The concrete molecular mechanism involved, specifically, whether HOXA11-AS plays pivotal roles in tumorigenesis and deterioration of NSCLC through regulating specific genes or as competitive endogenous RNA for specific miRNAs, still requires further elucidation through functional experiments.

## Conclusions

Our findings demonstrate that HOXA11-AS plays a significant role in NSCLC proliferation, invasion, migration, apoptosis and cell cycle. We also found that the HOXA11-AS was associated with tumorigenesis and angiogenesis in NSCLC. We speculate that HOXA11-AS may play a significant role in NSCLC carcinogenesis and progression by modulating the non-small cell lung cancer pathway. However, the exact molecular mechanism should be verified by future functional experiments.

## Materials and Methods

### Evaluation of the clinical value of HOXA11-AS in NSCLC

#### Significance of HOXA11-AS in NSCLC based on TCGA data

The Cancer Genome Atlas (TCGA) (http://cancergenome.nih.gov) database is one of the largest available public platforms, providing genomic, transcriptomic, methylomic and copy number variation (CNV) data sets for more than 20 cancer types^45–48^. TCGA can also be used to further research the complicated cancer genomics and various clinical parameters^49, 50^. In the current study, we downloaded the original data of RNASeqV2 in NSCLC, which consists of lung adenocarcinoma patients (535 lung adenocarcinoma cases vs 49 non-cancerous lung cases) and lung squamous cell carcinoma patients (502 lung squamous cell carcinoma cases vs 49 non-cancerous lung cases) from TCGA database (March, 31, 2017). Then the patients with the expression of HOXA11-AS less than 1 were excluded and the expression of HOXA11-AS was normalized by log2 transformed for the further analysis. Then, the expression of HOXA11-AS and the corresponding clinical parameters of the patients were extracted.

#### Quantitative real-time PCR

A total of 20 NSCLC cases, between January 2015 and August 2016, were collected from the Department of Pathology, First Affiliated Hospital of the Guangxi Medical University (Nanning, Guangxi, China). The average age of these patients was 55 years old (ranging from 38 to 79 years). All these cases were selected randomly from cases of surgical resection without treatment. All methods were carried out in accordance with relevant guidelines and regulations. And all experimental protocols were approved by the Ethical Committee of the First Affiliated Hospital of Guangxi Medical University, and consent forms were signed by the clinicians and patients for the use of the tissues for study. All the samples were confirmed by two independent pathologists who did not know the detailed patient information.

Total RNA was extracted using a Takara PrimeScript RT reagent Kit (Code: 9767) according to the manufacturer’s instructions. The extracted RNA was used for complementary DNA synthesis with a Takara PrimeScript RT Reagent Kit (Code: RR047A) according to the kit instructions. Then, qRT-PCR was performed using a LightCycler 480 Real-time PCR System (Roche, ShangHai). The primer pairs applied for PCR were as follows: HOXA11-AS, 5′-CGGCTAACAAGGAGATTTGG-3′ (forward) and 5′-AGGCTCAGGGATGGTAGTCC-3′ (reverse); GAPDH, 5′-ACCCACTCCTCCACCTTTG-3′ (forward) and 5′-CTCTTGTGCTCTTGCTGGG-3′ (reverse). One PCR cycle included 95 °C for 10 seconds, 60 °C for 20 seconds, 72 °C for 20 seconds. Expression data were normalized to GAPDH to guarantee stability of the expression levels. The relative value of HOXA11-AS expression was standardized using the formula of 2^−ΔΔCt^.

### Exploration of the effect of HOXA11-AS on biological processes in NSCLC

#### *In Vitro* experiments

Cell culture and Transfection: The human NSCLC cell lines A549, H460, 1299 and PC9 were purchased from the Type Culture Collection of the Chinese Academy of Sciences, Shanghai, China. All the NSCLC cell lines were cultured with 10% heat-inactivated fetal bovine serum (Invitrogen Corp, Grand Island, NY, USA) under 5% CO_2_ atmosphere with 2 mM gentamicin at 37 °C. The exponentially growing cells were used for the following experiments. For transfection, an effective shRNA targeting to HOXA11-AS was cloned into the plasmids on the base of vector backbone, GV248 and lentivirus-mediated HOXA11-AS RNAi was constructed. Three paired HOXA11-AS-specific shRNAs (GenePharma, Shanghai, China, Table 5) were synthesized and transfected into NSCLC cell lines to silence HOXA11-AS expression^51^. NSCLC cell lines, including A549, H460, H1299 and PC9, were transfected with lenti-HOXA11-AS RNAi or lenti-control virus to obtain the stable low HOXA11-AS-expressing cell lines. Then, 3 groups were designed in each cell line: blank control, lenti-control virus group (Negative control) and lentivirus-mediated HOXA11-AS RNAi group. Blank control groups were treated with only transfection reagent. Lenti-control virus groups were transfected with lenti-control virus (GenePharma, ShangHai). The Lipofectamine™2000 (Invitrogen, 11668–019) was applied for the transfection. In addition, after incubation for 72 h, puromycin (5 ug/ml) was added to select stable cell lines after transfection of shRNA plasmid. Then the transfection effciency was determined under fluorescence microscope and RT-qPCR.

**Table 5:** The sequences of HOXA11-AS shRNAs.

ID	Target Seq	GC%
HOXA11-AS-RNAi(32154-2)	CTACCATCCCTGAGCCTTA	52.63%
HOXA11-AS-RNAi(32155-1)	TGACATCCGAGGAGACTTC	52.63%
HOXA11-AS-RNAi(32156-1)	CGTAATCGCCGGTGTAACT	52.63%
lenti-control virus	TTCTCCGAACGTGTCACGT	52.63%

Cell proliferation assay: Cell proliferation was assessed by a cytometry assay and a CCK-8 assay (Dojindo, Japan). For the cytometry assay, the cells were seeded into 6-well plates with the cell density adjusted to 1 × 10^4^/well, whereas in the CCK-8 assay, the cells were seeded into 96-well-plates. Then, the well-plates were incubated at 37 °C in 5% CO_2_. Afterward, an automated count of the number of cells in the 6-well-plates was performed for 7 days. However, CCK-8 reagent was added to the 96-well-plates and the absorbance of each well at 450 nm was measured with a microplate spectrophotometer (KHB, Shanghai) at 12 h, 24 h, 48 h, 72 h and 96 h.

Cell scratch assay: Exponentially growing cells were seeded into a 6-well-plate. The seeded cells were cultured under a humidified atmosphere of 5% CO_2_ at 37 °C for 24 h, 48 h, 72 h and 96 h. When the cells spread to more than 80% of the 6-well-plate, 3 vertical lines were made in each well with a sterile 10 µL pipette. The migration distance was measured by Image-Pro Plus 6.0 according to the manufacturer’s instructions. The detailed distance was calculated by converting the pixels to millimeters.

Migration and invasion assay: To further research the effect of HOXA11-AS in migration and invasion of NSCLC cells, Transwell migration and invasion assays were carried out. Each cell line was divided into three groups (blank control group, lenti-control virus group and HOXA11-AS RNAi group). For the migration assay, 3 × 10^4^ cells were seeded into the upper portion of Transwell chambers (BD Bioscience), whereas for the invasion assay, the upper chamber was pre-coated with Matrigel (BD Bioscience) prior to adding the cells (3 × 10^4^). In both assays, the seeded cells were cultured for 24 h at 37 °C with 5% CO_2_. The cells on the filter surface were stained with 0.5% crystal violet and observed under a microscope. At least five random microscopic fields (200 × ) in each group were counted independently by two people. Then, the mean value was calculated.

Cell cycle and apoptosis assay: Cells were cultured in 6-well-plates. After 48 h, the cells were washed twice with phosphate-buffered saline (PBS) and then digested by trypsin. A flow cytometry assay was used to detect cell cycle phase and apoptosis. An Annexin V-PE/7-ADD Apoptosis Detection Kit (eBiosciences, USA) was utilized according to the manufacturer’s instructions. Additionally, the cell cycle phase distribution was determined, and the average number of dead and apoptotic cells was then compared to control groups.

### *In Vivo* experiments with a CAM model of NSCLC

Fertilized chicken eggs were obtained from Nanning Chicken Farm. Eight days after being hatched in an incubator, the embryos were evaluated for viability by trans-illumination of the egg in a dark room to identify the embryo and surrounding blood vessels^52, 53^. A one cm^2^ window was drawn on the egg shell overlying the most vascularized area of each viable embryo. Then, exponentially growing cells with different treatments were seeded in the embryo. Five days after inoculation, new blood vessels were generated, and the tumor xenografts were carefully removed and weighed. Then, the neo-vascular area was calculated by Image-Pro Plus software to evaluate tumor angiogenesis. In addition, the paraffin sections of tumor xenografts were observed under a confocal microscope.

#### The potential pathways associated with HOXA11-AS

To further analyze the potential pathways associated with HOXA11-AS, we used an open-access resource, Multi Experiment Matrix (MEM, http://biit.cs.ut.ee/mem/index.cgi)^21, 22^, to interactively explore the co-expressed genes for HOXA11-AS based on an Affymetrix Gene Chip Human Genome U133 Plus 2.0 Array platform. Then, functional enrichment analyses at the GO and KEGG pathway levels were conducted to infer HOXA11-AS co-expressed gene function by respectively using the DAVID Bioinformatics Tool (https://david.ncifcrf.gov/, version 6.7)^23, 24^ and KOBAS 2.0 (http://kobas.cbi.pku.edu.cn/)^54^. Both the GO enrichment and KEGG pathway analysis^55–57^ were performed by Fischer’s exact test. In this process, three independent categories derived from the GO analysis were included: biological process (BP), cellular component (CC) and molecular function (MF).

### Statistical analysis

All statistical analyses were performed using Statistical Package SPSS 22.0. In addition, comparison between two samples was analyzed by Student’s t-test. Comparison between groups was performed using one-way analysis of variance (ANOVA). For non-normally distributed variables, we performed Mann-Whitney U test and summary statistics were expressed as the mean ± standard deviation (Mean ± SD). The relationships between HOXA11-AS expression and the clinicopathological parameters were assessed by Spearman’s rank correlation. The clinical diagnostic value of HOXA11-AS was analyzed by a ROC curve. Differences in the survival curve were evaluated using a log-rank test and drawn using Kaplan–Meier method. P values less than 0.05 were considered statistically significant. In addition, all experiments were performed in triplicate.

### Statement

All methods were carried out in accordance with relevant guidelines and regulations. And all experimental protocols were approved by the Ethical Committee of the First Affiliated Hospital of Guangxi Medical University. Patients provide informed consent authorizing the use of their personal information and tissues for research purposes. Also, this study was fully compliance with the publication guidelines provided by TCGA and the approval of ethics committee was not required.
